# Boundaries and e-health implementation in health and social care

**DOI:** 10.1186/1472-6947-12-100

**Published:** 2012-09-07

**Authors:** Gerry King, Catherine O’Donnell, David Boddy, Fiona Smith, David Heaney, Frances S Mair

**Affiliations:** 1 Centre for Rural Health, University of Aberdeen, Centre for Health Sciences, Inverness, UK; 2 General Practice and Primary Care, Institute of Health & Wellbeing, University of Glasgow, Glasgow, UK; 3 Department of Management, University of Glasgow, Glasgow, UK

## Abstract

**Background:**

The major problem facing health and social care systems globally today is the growing challenge of an elderly population with complex health and social care needs. A longstanding challenge to the provision of high quality, effectively coordinated care for those with complex needs has been the historical separation of health and social care. Access to timely and accurate data about patients and their treatments has the potential to deliver better care at less cost.

**Methods:**

To explore the way in which structural, professional and geographical boundaries have affected e-health implementation in health and social care, through an empirical study of the implementation of an electronic version of Single Shared Assessment (SSA) in Scotland, using three retrospective, qualitative case studies in three different health board locations.

**Results:**

Progress in effectively sharing electronic data had been slow and uneven. One cause was the presence of established structural boundaries, which lead to competing priorities, incompatible IT systems and infrastructure, and poor cooperation. A second cause was the presence of established professional boundaries, which affect staffs’ understanding and acceptance of data sharing and their information requirements. Geographical boundaries featured but less prominently and contrasting perspectives were found with regard to issues such as co-location of health and social care professionals.

**Conclusions:**

To provide holistic care to those with complex health and social care needs, it is essential that we develop integrated approaches to care delivery. Successful integration needs practices such as good project management and governance, ensuring system interoperability, leadership, good training and support, together with clear efforts to improve working relations across professional boundaries and communication of a clear project vision. This study shows that while technological developments make integration possible, long-standing boundaries constitute substantial risks to IT implementations across the health and social care interface which those initiating major changes would do well to consider before committing to the investment.

## Background

A major problem facing health and social care systems globally today is the growing challenge of an elderly population with complex health and social care needs. A longstanding challenge to the provision of high quality, effectively coordinated care for those with complex needs has been the historical separation of health and social care, generally involving separate budgets for each, which is prevalent in many health care systems [1].

In order to provide holistic care there is a need to develop integrated approaches to health and social care delivery. Policymakers have realised that e-Health initiatives, and electronic record systems in particular, may help promote information sharing. Information Technology (IT) makes it possible for those delivering health and social care to exchange patient data without being constrained by time and distance. Access to timely and accurate data about patients and their treatments has the potential to deliver better care at less cost [2,3]. However, many local successes are not extended to a national scale or integrated with other parts of the health or social care system [4-6], implying that much IT spending is wasted.

For example, in the US, Stead and Lin [6] found that most health care IT systems were poorly integrated into clinical practice, rarely being used to support clinical decisions or to provide an integrated view of a patient [6]. The research team concluded that this was due to several factors, including: ignoring well-known principles of human-computer interaction; failing to change working processes to accommodate the system; and using monolithic systems which were hard to adapt to local conditions. The research showed how general organisational factors had prevented the effective use of many IT systems, but did not consider which practices might support more effective implementation. Such knowledge may enable health care providers to develop their capability to deliver appropriate health care IT systems by combining technical resources and organizational competence.

Stead and Lin also identified problems caused by the fragmented US health care system in which the many players have their own rules, priorities and incentives [6]. This theme is addressed empirically in a European context by Boonstra et al. who found that implementing an inter-organisational system requires significant organizational as well as technical changes [7]. These changes affect stakeholders and system users (upon whom implementers depend) who have varying degrees of power and interest in the system. Identifying stakeholders and understanding their attitudes and needs will enable implementers to meet their expectations more fully, and so encourage acceptance. The authors examined these issues by studying the introduction of an Electronic Patient File system in The Netherlands. All involved believed the system would benefit patients, yet powerful players resisted its implementation, fearing it would affect their interests. Those with high interest in the system lacked the power to implement it, while those with low interest in the proposed system had the power to block it. These negative attitudes were shaped by concerns not about the system itself, but about the likely effects on that profession’s working routines, power, culture and financial arrangements. Thus, system implementers should seek to identify and reconcile stakeholder interests.

Research shows that promoters of information-sharing projects consistently underestimate the changes required to successfully implement IT-based care projects [8-10]. These changes are difficult to manage in a single unit, such as a single healthcare team; the risks increase when, as in health and social care, staff from different organizations and professional backgrounds need to work co-operatively using shared IT systems to exchange and use information across boundaries [7]. Such boundaries might be professional, structural or geographical. Projects which try to cross or challenge such boundaries are inherently high risk, yet health care systems internationally are actively addressing the integration of primary, hospital and community-based care [11-13].

The concept of boundaries is well established [14-16]. Many writers see professional boundaries as a major influence on organisational practice, and the ease with which change is accepted. As health and social care providers are highly professionalized institutions, we decided to study the effect of this form of boundary on the implementation of a change intended to enable data-sharing [17]. The effect of structural boundaries was included since the structural division of the broad task of ‘care’ into distinct ‘health’ and ‘social’ components is fundamental to attempts to change the delivery of care: the capacity of governments to address issues that require co-ordinated action is limited by the structure of their systems [18]. For example, the United Kingdom (UK) welfare state has divided responsibility for delivering health and social care, with each sector reporting to different ministers, health care or local government committees. This kind of separation promotes the development of unique internal structures, cultures [19], ways of working [20], financial and information systems [21]. Finally, policy makers and practitioners have recognised that geographical location can also act as a “boundary” [22]. Geographical boundaries are of particular interest in a country such as Scotland where physical distances can impede access to care, while at the same time technology is often seen as a way of overcoming this barrier to care delivery. At an early stage of the study we considered including ‘functional’ boundaries in the interview schedule, but found that it became entangled with the concept of ‘structural’ boundaries, and so excluded it from the research.

Professionals have been shown to value control over boundaries and territory, while also recognising their fluid nature [17]. Currie et al. point out that many proposals for UK public service reform depend on new ways of managing knowledge across organisational and professional boundaries [23]. They pay particular attention to the role of professional cultures as a significant factor in knowledge sharing. Managing these cultural differences to secure positive outcomes for users of a public service depends on a deep understanding of the nature and formation of these cultures-especially when, as in many parts of the NHS these are fragmented and self-contained sub-cultures. Their empirical study in a university teaching hospital showed how professional groups perceive the world from diverse perspectives, which has a significant bearing on their views about information-such as what events are worth recording or what information should be shared with other professions. Many of these behaviours were rooted in a common mistrust of other professionals, and that ‘As a consequence of socialization along narrow professional lines, different occupational communities appear ill-equipped to cope with the challenge of working across professional boundaries’ [23] (p.381).

It is clear from accumulating evidence such as this that implementing an inter-organisational information system requires providers of health and social care to develop a widespread capability amongst their staff to implement such changes. This paper contributes to the evidence base by examining the very specific ways in which boundaries affected behaviour and consequently the integration and embedding of a shared electronic health record.

Our aim was to explore the way in which structural, professional and geographical boundaries have affected e-health implementation in health and social care, through an empirical study of an electronic version of Single Shared Assessment (SSA) in Scotland.

SSA is part of a Scottish Government strategy to improve community services for users by redesigning the assessment system that people go through at home when they need to access those services [24]. Thus, previously separate systems in finance, management and the delivery of services are being brought together to provide a more holistic approach to service delivery, with the aim of streamlining and speeding up the process of assessing an individual’s need for services. SSA is designed to benefit the people who use the services, the agencies and the professionals who deliver the services. SSA aims to be person-centred and led by a single professional with other specialist involvement as appropriate. So, where as previously there would be different assessments done by different professionals at different times, now there should be one assessment conducted by the most appropriate professional and that information shared, if the patient gives consent. People who use the services and their carers are intended to be at the heart of the assessment process. However, to ensure the most appropriate assessment response, agencies need an integrated system for receiving and acting on referrals. Ideally this should be done through the use of a shared electronic record. Health Boards (responsible for health) and local authorities (responsible for social care) were tasked by the Government with implementing the SSA, mainly through District Nurses, Allied Health Professionals (AHPs) and Social Workers.

## Methods

### Study design and setting

We used a retrospective, qualitative case study approach to examine how structural, professional and geographical boundaries affected the implementation of an electronic SSA. Case studies have been described as an exploration of a bounded system, for example bounded by time, place or system [25]. Here they were defined and bounded by the SSA projects whose implementation and use we studied in three extensive geographical locations in Scotland, each the responsibility of a different health board. We chose the three sites (out of a possible 14) on the basis of (1) our previous research [26,27]); (2) personal communication; and (3) pragmatic requirements of time and funds. The first case is located in an NHS region working with one Local Authority Council, with significant remote and rural issues. The second case is located in a smaller, more urban NHS region, but interacting with three local authorities. Finally the third has one NHS region and one council but covers a large geographical area, with some rural areas. These three cases were significant because of their variation in size and geographical challenges. Comparing these three case study sites was especially relevant because one site was anecdotally more successful in terms of implementation of the electronic SSA than the others. The research team decided not to publish the identity of the sites as it would then make it easier to identify individuals within those sites and compromise the anonymity we had assured participants. This study was approved by the Glasgow University Medical Faculty Ethics Committee as approval from NHS Research Ethics Committee was not required.

### Data collection and analysis

#### Interview data

We interviewed 30 health and social care professionals across the three sites. We held scoping discussions with key stakeholders to identify professional groups using (or meant to be using) SSA, and key managers and implementers. The design of the interview schedule (Additional file 1) was informed by the e-health model being implemented and by our earlier work [27]. The interview schedule was ‘tested’ in a pilot interview. The schedule was not revised following the pilot because we found that it worked well.

In order to make conceptual rather than statistical generalisations, we included a wide range of professionals involved with SSA in the sample and conducted semi-structured face-to-face interviews (Table 1).

**Table 1:** Affiliations and job titles of interviewees

**Area 1**		**Area 2**		**Area 3**	
NHS staff (five, including community and hospital nurses, AHPs, managers)	Local authority staff (five, including social work care assessors and managers)	NHS staff (six, including community nurses, AHPs, managers)	Local authority staff (five, including social workers and managers)	NHS staff (five including community nurses, and IT staff)	Local authority staff (four including social workers)

Footnote: individual disciplines have not been identified to protect anonymity.

### National telephone survey

We also conducted telephone interviews with the data sharing manager (DSM) responsible for promoting cross-boundary information exchange in each health board area across Scotland, to allow them to describe progress in their respective areas, thus allowing us to relate the case study evidence to the national context. We invited DSMs (or similar) from the 14 board areas to take part, and 11 areas responded (n = 11 interviews).

### Data analysis

Interviews were recorded and transcribed, with informed consent; analysis was informed by Framework Analysis [28]. The coding schedule was developed by the team (Additional file 2). Five members of the team independently coded five transcripts, and held a ‘coding clinic’ to develop the coding index ensuring the reliability of our inferences. The qualitative data analysis program ‘Nvivo’, was used to organise the data. All interviews were double-coded and the whole team iteratively reviewed the coding schedule during the course of the study to ensure all key issues were captured and accurately conceptualised.

### Presenting the data

We present our results as answers to four questions which follow directly from the study aim and our theoretical focus on the role of boundaries in affecting the implementation of SSA. The first question asks about the extent to which the 11 areas responding to the national telephone survey had implemented SSA, while those which follow present the evidence on the effect of structural, professional and geographical boundaries respectively. Within each question, we identify by sub-headings the most prominent factors to emerge from our analysis of the data associated with that boundary.

## Results

### What outcomes did stakeholders expect from SSA, and to what extent were they achieved?

SSA was meant to create a holistic record of the patient’s condition and circumstances, which health or social care staff could use to assess care needs. It was expected to reduce the number of times patients were asked for the same information by different professionals, reduce the duplication of records and increase accuracy of information thereby leading to improved quality of care.

We found that progress towards these outcomes could at best be described as ‘limited.’ All areas had adopted paper SSAs, though in several cases these were rarely used, as the normal method was to record the assessments electronically. All but one area used electronic SSAs-though the extent to which this happened varied between locations within the area-some were almost completely electronic, others were still in the process of implementing them. On the key question of the extent to which staff were sharing the assessments electronically (the main intended outcome of the project), answers were mixed, with the 11 DSMs interviewed answering: yes-2 sites; limited-6 sites; no-3 sites. One of the two DSMs whose colleagues were sharing data electronically explained: ‘it has increased sharing compared with paper, as it is much easier to use’. One, in an area where sharing was limited, said ‘other professions can’t add [information] to the SSA, so they send it to social work who enter it’, while one where there was no sharing explained that this was due to ‘the technical challenges of three computer systems and the cultural differences between health and social work’.

As one respondent, who had initially made good progress commented, momentum was now being lost:

‘Management don’t appear to want to get the information. I think staff are feeling that nobody’s interested, so why bother? It’s affected by the eCare framework, which has failed to materialise, so there has locally been a reluctance to maintain what we’ve got, just in case the national one came along’. (DSM1)

A similar pattern was evident in our three case study sites. While one had made more progress than the others, the sense was of a project which had not yet achieved the outcomes expected, and which many felt were now unlikely to be achieved. One respondent said:

‘I don’t think it has had the impact it was meant to. I’d give it about 1 out of 10. I don’t think it has helped in the slightest. We don’t share a lot of them… … they’re probably not a particularly good assessment either. In terms of a joint document it’s pretty pointless really’. (Interviewee 3-health)

Staff were aware of both the positive and negative outcomes of SSA-commenting for example on the advantages of having an electronic record or, elsewhere, on the disadvantages of the bulky paper document. There were also many comments about how staff used SSA-sometimes as a full shared assessment as intended, by others as a tool for securing a specific care service for a patient. Units also varied in the extent to which they stored the assessments on paper or electronically. A common theme was that the SSA would not be implemented successfully until adequate IT systems underpinned the document.

### How did structural boundaries affect electronic data sharing?

The structural boundaries likely to be most relevant are those delineating the delivery of health and social care respectively. NHS Scotland delivers services through 14 Health Boards which, though funded by the Scottish Government, have considerable autonomy over the way in which they deliver them. They can decide how much to spend on information systems, and which systems they want to use or develop. Within Boards, each hospital or clinic can also decide on the disposition of IT expenditure-even where national systems are developed by the centre, units can decide whether or not they use them. Care services are delivered by 35 local authorities which, though substantially funded by the Scottish Government, have a high degree of autonomy over policy, including IT provision.

### Competing priorities

Health boards and local authorities face competing demands for support from those promoting the many services they provide, as does the Scottish Government which supports them. Staff in both areas talked about the difficulties of different services having their own priorities and information needs, for example to monitor their service in relation to national and local performance targets. They also questioned the Scottish Government’s commitment to the SSA and what their priorities are:

‘I’ve a feeling that the Scottish Government’s starting to move away from it (SSA), because we’ve now switched to this enablement model and more of an outcome focused model of working …so I think this, this dawn of the SSA might not . continue … this is about 10 years on and we’re still struggling’. (Interviewee 19-social work)

Competing priorities were described on an individual, professional, organisational and governmental level and were seen as a major problem.

‘… unfortunately they’ve got ministers saying no you need to deliver on this, this and that, and that’s a priority and that’s not. I think SSA is probably left to us to get on with now … But … we need national support. We need national networks where … anybody that has got some sort of remit for SSA, you can share experience of good practice or bad practice, or just having a sounding board and because it’s a very isolating place to be’. (Interviewee 22, management)

### IT systems and infrastructure

There are significant differences in IT infrastructure between health and local authority organisations. Local authorities have one system, called CareFirst, that can be easily connected to the eCare framework, but NHS Scotland, with multiple IT systems, cannot. Respondents talked about how clumsy and time consuming it was to complete the SSA on paper and expressed frustration that the technology could not support the successful implementation of the SSA.

One respondent noted that health and social care appeared to be at different stages in the use of IT as well as the infrastructure:

‘Probably the biggest [obstacle] is the lack of IT infrastructure in health. Most social workers were using computers regularly, often laptops, mobile devices. This was just a new application we had to teach them how to use. A lot of the health professionals we dealt with hadn’t come across mobile devices before. I think they found it in some ways quite challenging’. (Interviewee 12-health)

Even if health boards and local authorities were at similar stages of IT use, these have developed independently, and so required an interface to link them. This interface was to be achieved through the ‘e-Care framework’, but several respondents claimed that this system was not able to meet the needs of users, and required significant work to put right:

‘The e-care framework can currently [only] allow a system to publish and view SSAs. Its implementation has been fraught with problems because of a change of government, a change in leadership, a lack of leadership and just a poor implementation plan from the Scottish Government’. (Interviewee 2-health)

The pressing need for more system interoperability was also a recurring theme.

‘So you have got systems that can’t link up, that won’t link up and I think there is a huge call for a system that did marry up’. (Interviewee 29, health)

Positive examples of sharing information were reported in spite of the lack of IT infrastructure, but it was widely acknowledged that data sharing would be much easier if it were supported by electronic systems.

### Financial arrangements

Implementing an electronic SSA has initial and ongoing financial implications. The Scottish Government provided £150,000 over three years to each area to implement the SSA electronically as part of the eCare system. Towards the end of the second year there was a change in government and funding was reduced. When that funding ceased, councils and health boards had to decide on their local priorities:

‘We were getting financial help from Scottish government to take forward SSA… That funding is no longer there so we’ve had to absorb it within our own organisations. That will have had an impact-there’s [part of] my post…and also we did have admin support and things. Whether it’s financial costs or just implications for staff having to try and catch up when they go back’. (Interviewee 4-social work)

Respondents suggested that future developments in IT may also depend on the willingness of local Health Boards and councils to provide funding; which might not be prioritised in times of financial constraints. Since 2009, there has been no funding at all dedicated to SSA with councils or health boards having to commit their own funding to taking this forward.

### How did professional boundaries affect electronic data sharing?

The professional groups most involved in SSAs are social workers (within which there are further specialities such as mental health) and the health professions (especially community nurses). Professional boundaries between these groups affected their respective understanding and acceptance of the aims of SSA, the information they require to do their jobs, and whether they see SSA as consistent with their culture.

#### History of co-operation

How people act is influenced in part by historical factors and, in this context, this means the extent to which they have worked collaboratively with colleagues in other agencies. There has been a long history of attempts to bring health and social care services closer together with limited success, as the following respondent highlights:

‘SSA was a naïve political vision [based on] the idea that we had a joint future, and as a joint future has never taken place, SSA has always struggled…we’ve never worked closely together particularly at the management level with our Social Work and Local Authority colleagues. [Barriers include] trust, budgets, management structures, councillors trying to control services, health board members not elected, not wishing to share funds’. (Interviewee 3-health)

This was a significant theme throughout the interviews with health and social work staff. Respondents talked about distinct professional boundaries being maintained by each agency with an acceptance that that’s the way it’s always been and that it is unlikely that it will change. The following interviewee gave a typical view:

‘I think there is still an underlying trend that, oh well that’s health and that’s social work and never the twain shall meet which is a real pity but that’s how staff are’. (Interviewee 4-social work)

Importantly, there was evidence that such barriers to joint working could be overcome. For example, one area instigated an initiative called ‘virtual teams’ which was a joint programme designed to improve communication and understanding of professionals’ roles. They used creative media to raise awareness of communication issues and professional boundaries. The following respondent talks positively about the scheme:

‘Because there were limited communications between the two groups of people and it was leading to a sort of blame culture well this is social work’s paperwork or, or health aren’t complying with this and that wasn’t the case, so they did a em, it was a like actors came in and did a wee scenario, but very comically showed us what we looked like in a sense, you know blaming each other and things and that was really useful as well, because everybody could relate to it and then we sat, sat down in group works and actually started to look at paperwork and look at how we used it and it was, it was really useful’. (Interviewee 17-social work)

### Understanding and acceptance of aims of SSA

There were a range of views about the aims of the SSA. Many described the aims in terms of theory and practice, explaining that they were different. Typically, staff would outline the theoretical aims of the SSA in terms of holistic care, joint working and reduced duplication and then talk about how it works in practice. In practice, many professionals were using the SSA as a referral document to secure services for patients. The following respondent articulated a common view:

‘…if you’re gonna get a service from social work then this is the ticket’. (Interviewee 8-health)

One manager wondered if the management team was partly responsible for staff perceiving the SSA as a referral document. Because the professionals were reluctant to use the SSA, managers made the decision to make it a necessary part of the referral process. The following comment describes the unintended consequence of this decision:

‘because the decision was made that you had to do a SSA to access other services, then it was never seen as, you know, the full assessment, it was seen as a way to get into another service as opposed to what it should have been …’ (Interviewee 12-social work)

While some who were responsible for the implementation of the SSA expressed their frustration with professionals, others did describe positive examples of how the aims of SSA were accepted yet still proving difficult to enact in practice.

‘I think it’s fairly well understood in theory. I’m not sure that they, they see it the same when they’re actually in practice and doing all these things but they see that yes, it’s useful. And they certainly come back to us when we’ve done training or when we’ve gone back out and yes it is a really good document but I don’t have time to do it, they’re just not seeing the whole picture. Em, so I mean it has fallen down as far as that goes where, yeah, the theory of it is great but in practice it’s just not hitting the mark’. (Interviewee 4-social work)

### Information requirements

Professions often have different beliefs about the information they need to do their work. All staff assess a system’s ability to meet their information needs by its usefulness and ease of use. Commonly practitioners found it neither useful nor easy to use:

‘I think they are not all that happy with the assessment because it is very time consuming and as I say it doesn’t always give us the results that we want to get’. (Interviewee 25, health)

Many of the health staff criticised the SSA document for not having enough clinical information and for covering more ‘social’ than ‘health’ information:

‘It’s more a social work document than a health document, and there’s still this huge gap between Social and Health’. (Interviewee 5-health)

Health staff also suggested that much of the information in the document was not important or not appropriate, for example, ethnic origin, religion and first language. There was dissatisfaction about the information needed to meet the Scottish Government minimum data standards, which was contrary to what practitioners think they need to give care to their patient. In addition to the type of information required, practitioners questioned whether it was necessary to undertake a full and often unwieldy assessment for what they considered a relatively simple need like a ‘helpcall’ which is a push-button device that they wear around their neck:

‘Why do a whole 14 page assessment when actually the person needs a helpcall? What is it we need to know about the person for them to have a helpcall? We don’t need to know all their background and how many times they’ve been married’. (Interviewee 8-health)

There was variation and some confusion about how much information assessors should collect. What they collected was dependent on many factors including personal choice and management support.

Finally, staff evaluate a system’s ability to provide them with information in terms of how it fits with established working processes-a close fit is likely to encourage readier acceptance than one which requires costly changes to present methods.

### Culture

Professions have different views about what is important in their work, what they value, and their willingness to share information. Health service professionals work in a system where delivery is free at the point of access-and are sometimes reluctant to conduct an assessment that supports the delivery of means-tested services:

‘The social care component is means tested-if you’ve got the money you pay for it-and ethically we’ve always gone for service being free at the point of access. Doing a financial assessment might affect a nurse’s relationship with their patients-asking to see their bank book, or asking how much their house is worth’. (Interviewee 3-health)

Social workers, in contrast, are familiar with a range of services whose delivery depends in part on someone’s income-so the SSA does not conflict with their values.

Another aspect of a culture is the willingness of members to share information. While it is fundamental to the success of the SSA there was some evidence that health service staff were more protective of the information they held than the local authority staff. Some of this appeared to be judicious but some staff projected a heightened sense of ownership and possession of information which was not easily understood as necessary for information governance. This may be more consistent with health service culture:

‘One reason we don’t add much detailed information to the form is that we don’t want to give out too much medical information-the perception is we don’t want to give out any more information than we have to’. (Interviewee 3-health)

Commonly, respondents from health and social work considered culture to have a significant impact on how well they worked together and how well the SSA was accepted and integrated as a joint document. Nurses were more likely to say that the SSA did not fit with their perception of their role, culture or the work they do:

‘When we were evaluating the new SSA, I had quite a lot of comments from nurses saying I will not be filling in this section around employability, house care and finances because I’m a nurse and that’s a social worker’s job’. (Interviewee 22-management)

### How did geographical boundaries affect electronic data sharing?

Geographical challenges were noted at all sites and had the potential to affect the ability to share information easily. The SSA was seen as a solution to overcome the challenges of joint working at a distance. Co-location, remote and rural practice and primary/secondary care settings emerged as significant issues.

### Co-location

The study sites had significant experience of working in integrated teams whereby members from several different disciplines worked in the same room. This was particularly useful for completing SSAs because practitioners could access each other’s computer systems in a way that was impossible to do otherwise. Commonly, practitioners were positive about working together in integrated teams as in the following case:

‘There were 30 of us all in the one room and so I found you were able to see some of them and you knew they were there and you could talk to them and sometimes they would come up and sit next to you and you would type a new SSA, you know, what the transfer was. I think all being in the one room was a big help’. (Interviewee 7-health)

Commonly, there were several SSAs completed for the same patient as practitioners were unaware if one had been started. Having access to each other’s systems meant that practitioners could check if an SSA had been started and locate it, if that was the case.

In two cases, practitioners had been working in co-located teams and were disappointed when the teams were disbanded as part of restructuring.

‘It’s quite a difficult time at the moment … the ICAS [Integrated Community Assessment Service] team is actually been disbanded at the moment so we’re going through quite a lot of change within social work’. (Interviewee 16-health)

### Remote and rural practice

Some practitioners did identify that joint working and sharing information was more successful in geographically defined places.

‘There was much more of a cohesive team, if you like, even although they weren’t maybe centred in the same place, but because of the nature of some of the rural areas for instance, all the professionals relied on each other to get something to happen. You didn’t have, you know, the choice of six or seven professionals to go to. You either knew who the social worker was in that area, you knew who the policeman is or the policewoman is, you knew the GP. So there was that kind of sort of hub, if you like, of people that worked really well together’. (Interviewee 8-social work)

In addition, there were issues around IT infrastructure and connectivity which were particularly pertinent in remote and rural areas as outlined in the following quote.

‘We have tried other pilots, … to do with tablets … and a lot of the time we just can’t get a decent enough signal to be able to transfer information via a wireless connection or a broadband connection’. (Interviewee 4-health)

### Primary/secondary care setting

Boundaries between primary and secondary care were evident in fundamental ways. In one of the areas, the practitioners were convinced that most of the staff in the main referring hospital would not have heard of an SSA. So staff in the hospital were neither initiating SSAs nor using them as sources of information on admission as explained in the following quote:

‘From a hospital point of view, we’d never used it. I’d heard of it and that was all. So going out in the community last year, when I worked with augmented care at home, it was very much a part of their, assessment and criteria. (Interviewee 1-health)

However, in other areas, the practitioners highlighted that it was easier to complete an SSA if you were working on the wards because the patient, relatives and professionals were all more accessible.

## Discussion

Our work has explored and highlighted the way in which structural, professional and geographical boundaries can impact on the implementation of an ehealth service intended to work across such boundaries. Cross sectoral IT services are intended to improve a range of outcomes but generally involve multiple stakeholders with different professional cultures, and competing interests. While the idea of electronic data sharing might appear to be an uncontentious way to improve patients’ experience of care and to reduce its cost, little progress has been made in implementing it. This is mainly due to the obstacles posed by the structural, professional and geographical boundaries we have described. Our findings are consistent with previous literature on the diffusion of innovations [29], and suggest that structural boundaries between health and social care staff shaped their respective priorities, IT systems and ability to cooperate. Similarly, professional boundaries affected their respective understanding and acceptance of the aims of SSA, as these boundaries influenced the information required to do their jobs, and whether they saw SSA as supporting or undermining their professional culture and ethos. These boundaries are however, not immutable forces of nature, but the result of specific, identifiable, actions and decisions-to change priorities, reallocate funds and maintain current working practices. There was also evidence of human action overcoming boundaries-as when some professionals chose to work together in a new way to improve service delivery, while colleagues in another area did not do so. These findings resonate with previous published literature [16,30] which has found that the contours of boundaries are shaped by local forces and are not necessarily rigid.

There were also issues of jurisdictional legitimacy, such as whether completion of the electronic SSA fits with nurses’ perception of their role. This is consistent with the findings of Motulsky [31] in relation to implementing IT systems that cross professional boundaries, and existing conceptual models such as Abbott’s [14] who argues that professional boundaries are in a state of perpetual dispute and re-alignment [14,31]. We have also demonstrated that it is essential to ensure that any cross-sectoral IT innovations meet sufficient interests of stakeholders across sectors to ensure their support, consistent with research findings in other spheres of health service delivery [32].

The use of semi-structured interviews, theoretically-grounded in our previous work, enabled respondents to describe their experience of SSA in their own terms, providing a rich set of interview transcripts. There has been a call for studies that explore process and impact in relation to the implementation of innovations in health service delivery [29] and we would suggest that this work contributes to addressing that gap. This study examines issues affecting the implementation of shared IT systems across health and social care through the lens of boundaries but incorporates socio-technical considerations as advocated by Berg et al. and issues of workability as proposed by May and others [10,33-35]. For example, our approach takes account of issues such as the degree of “fit” of the new system with existing work practices, ease of use of the system, and how individuals make sense of and appraise the innovation thereby demonstrating potential synergies of this perspective with previous work.

One limitation of this study is that we provide a snapshot rather than longitudinal data. Thus we have not, within this study, been able to track possible progress towards implementation. Another limitation is that, this study describes the experience of one country. Nevertheless, we believe the research has given reliable and generalisable insights into the factors helping and hindering electronic data sharing between health and social care.

Our study is consistent with the stream of work in organisational theory stemming from Pettigrew’s [36] paper, which drew attention to the significance of internal, external and historical contexts for human action [36]. We have shown that structural boundaries present substantial risks to endeavours to encourage data sharing between health and social care. Boundaries are not mechanical divisions which can be crossed easily by appropriate information systems. Rather the units which boundaries create take on independent lives, as staff within them develop priorities which meet the expectations of their immediate stakeholders-health services and local government respectively-as well as the many autonomous units within each sector. In the present context, these units in health and social care developed independent IT systems and resource allocations which make cross-boundary data sharing difficult-especially when the agencies do not generally have a history of close cooperation.

It is also consistent with theories about the influence of professional boundaries [23], showing how these hindered progress-not because individual practitioners are unwilling to use new systems, but because of deep-seated differences in professional values [23]. These shape the information that respective professionals need to do their work; how easily the provision of, or access to, this information relates to established working processes; and whether their culture focuses on the patient’s immediate clinical condition or their wider social context.

### Conclusions

Managing these risks requires implementation processes at both national and local levels which are up to the task in areas such as identifying stakeholders, project management and governance structures, which can ensure interoperability of systems, a common understanding and acceptance of project aims, and the communication of the project’s vision to those involved. This resonates with recent literature which emphasizes the importance of engagement of stakeholders in the development and implementation of e-health systems [37,38]. There were several examples of good practice on these points-but these were essentially local or isolated initiatives, not part of a wider and sustained implementation plan to ensure delivery of the SSA as envisaged.

Importantly, our work identifies potential solutions to these problems such as recognition of the necessity for adequate project management of those aspects of the project which reside at national level; adequate project management capability at the level of each partnership to deliver and sustain the substantial organisational changes required. Quality leadership, and good training and support, together with clear efforts to improve working relations between professionals from different backgrounds will also make a difference.

The key message, however, is that long-standing boundaries constitute substantial risks to IT implementations across the health and social care interface which those initiating major changes would do well to consider before committing to the investment.

## Competing interests

The authors have no conflicts of interest to declare.

## Authors’ contributions

FM was PI and COD, GK, DH, DB were co-investigators on the grant that funded this research. GK, DB, and FS undertook primary data collection. All authors contributed to development of the coding schedule and coding of transcripts. FM, GK, DB, and COD led drafting of this paper which was reviewed and commented upon by all authors. All authors read and approved the final manuscript.

## Pre-publication history

The pre-publication history for this paper can be accessed here:

http://www.biomedcentral.com/1472-6947/12/100/prepub

## Supplementary Material

Additional file 1 Interview Schedule. Click here for file

Additional file 2 Coding scheme for CSO boundaries project. Click here for file

## References

[B1] Weiner K Hughes J Challis D Pedersen I Integrating health and social care at the micro level: health care professionals as care managers for older people Soc Pol Admin 2003 37 5 498 515 10.1111/1467-9515.00354

[B2] Davis K Slowing the growth of health care costs: learning from international experience New Eng J Med 2008 359 1751 1755 10.1056/NEJMp0805261 18946060

[B3] Mongan JJ Ferris TG Lee TH Options for slowing the growth of health care costs New Engl J Med 2008 358 1509 1514 10.1056/NEJMsb0707912 18385503

[B4] House of Commons Health Committee The Use of New Medical Technologies within the NHS. Fifth Report of Session 2004-05 2005 Available at: www.publications.parliament.uk/pa/cm200405/cmselect/cmhealth/398/39802.htm. Accessed 31 August 2012

[B5] Currie WL Guah MW IT-enabled healthcare delivery: the U.K. national health service information systems management Inform Syst Manag 2006 23 2 7 22 10.1201/1078.10580530/45925.23.2.20060301/92670.3

[B6] Stead WW Lin HS Computational technology for effective health care 2009 National Academies Press, Washington, D.C 20662117

[B7] Boonstra A Boddy D Bell S Stakeholder management in IOS projects: analysis of an attempt to implement an electronic patient file Eur J of Info Syst 2008 17 2 100 111 10.1057/ejis.2008.2

[B8] Boddy D Boonstra A Kennedy G Managing information systems: Strategy and organisation 2009 3 Financial Times/Prentice Hall, Harlow

[B9] Robertson A Cresswell K Takian A Petrakaki D Crowe S Cornford T Implementation and adoption of nationwide electronic health records in secondary care in England: qualitative analysis of interim results from a prospective national evaluation BMJ 2010 341 c4564 10.1136/bmj.c4564 20813822 PMC2933355

[B10] Murray E Burns J May C Finch T O’Donnell C Wallace P Mair F Why is it difficult to implement e-health initiatives? A qualitative study Implement Sci 2011 19 6 6 21244714 10.1186/1748-5908-6-6 PMC3038974

[B11] Rittenhouse DR Shortell SM The patient-centered medical home: will it stand the test of health reform? JAMA 2009 301 19 2038 2040 10.1001/jama.2009.691 19454643

[B12] Stange KC Miller WL Nutting PA Crabtree BF Stewart EE Jaen CR Context for understanding the national demonstration project and the patient-centered medical home Ann Fam Med 2010 8 1 52 58 10.1370/afm.1110 PMC2885730 20530391

[B13] Commonwealth of Australia Improving primary health care for all Australians 2011 Australian Government, Canberra

[B14] Abbott A The system of professions: An essay on the division of expert labor 1988 University of Chicago Press, Chicago

[B15] Allen D The nursing-medical boundary: a negotiated order? Soc Health Illness 1997 19 4 498 520

[B16] Mizrachi N Shuval JT Between formal and enacted policy: changing the contours of boundaries Soc Sci Med 2005 60 7 1649 1660 10.1016/j.socscimed.2004.08.016 15652695

[B17] Jones I The theory of boundaries: impact on inter-professional working J Inter-prof Care 2007 21 3 355 357 10.1080/13561820701257383 17487712

[B18] Sullivan H Skelcher C Working across boundaries: Collaboration in public services 2002 Palgrave Macmillan, Basingstoke

[B19] Boonstra A Boddy D Fischbacher M The limited acceptance of an electronic prescription system by general practitioners: reasons and practical implications New Tech Work Employ 2004 19 2 128 141 10.1111/j.0268-1072.2004.00132.x

[B20] Mellin A E-prescribing: an opportunity for process-re-engineering Health Management Technology 2002 43 1 42 47 11808478

[B21] Rummery K Coleman A Primary health and social care services in the UK: progress towards partnership? Soc Sci Med 2003 56 8 1773 1782 10.1016/S0277-9536(02)00173-9 12639593

[B22] Exworthy M Peckham S The contribution of coterminosity to joint purchasing in health and social care Health Place 1998 4 3 233 243 10.1016/S1353-8292(98)00018-5 10670974

[B23] Currie G Waring J Finn R The limits of knowledge management for UK public sector modernization: the case of patient safety and service quality Publ Admin 2008 86 2 363 385 10.1111/j.1467-9299.2007.00705.x

[B24] Executive S Guidance on single shared assessment of community care needs-circular CCD 8/2001 2001 Scottish Executive, Edinburgh

[B25] Creswell JW Qualitative inquiry and research design. Choosing among five traditions 1998 Sage Publications, London

[B26] Mair F May C Murray E Finch T O’Donnell CA Sullivan F Anderson G Wallace P Understanding the implementation and integration of e-health services 2009 Report for the NHS service and delivery R & D Organisation (NCCSDO), London

[B27] Boddy D King G Clark JS Heaney D Mair F The influence of context and process when implementing e-health BMC Med Informat Decis Making 2009 9 9 10.1186/1472-6947-9-9 PMC2642812 19183479

[B28] Ritchie J Spencer L Qualitative data analysis for applied policy research. Analyzing Qualitative Data 1994 Routledge, London

[B29] Greenhalgh T Robert G Macfarlane F Bate P Kyriakidou O Diffusion of innovations in service organizations: systematic review and recommendations Mil Quart 2004 82 4 581 629 10.1111/j.0887-378X.2004.00325.x PMC2690184 15595944

[B30] Shuval J Nurses in alternative health care: integrating medical paradigms Soc Sci Med 2006 63 7 1784 1795 10.1016/j.socscimed.2006.05.009 16781808

[B31] Motulsky A Sicotte C Lamothe L Winslade N Tamblyn R Electronic prescriptions and disruptions to the jurisdiction of community pharmacists Soc Sci Med 2011 73 1 121 128 10.1016/j.socscimed.2011.04.009 21664019

[B32] Haddow G O’Donnell CA Heaney D Stakeholder perspectives on new ways of delivering unscheduled health care: the role of ownership and organizational identity J Eval Clin Pract 2007 13 2 179 185 10.1111/j.1365-2753.2006.00667.x 17378862

[B33] Berg M Aarts J van der Lei J ICT in health care: socio-technical approaches Methods Inform Med 2003 42 4 297 301 14534625

[B34] May C Finch T Implementation, embedding, and integration: an outline of normalization process theory Sociology 2009 43 3 535 55

[B35] Murray E May C Mair F Development and formative evaluation of the e-health implementation toolkit (e-HIT) BMC Med Informat Decis Making 2010 10 1 61 10.1186/1472-6947-10-61 PMC2967499 20955594

[B36] Pettigrew A Context and action in the transformation of the firm J Manag Stud 1987 24 6 649 670 10.1111/j.1467-6486.1987.tb00467.x

[B37] May CR Finch TL Cornford J Exley C Gately C Kirk S Jenkings KN Osbourne J Robinson AL Rogers A Wilson R Mair FS Integrating telecare for chronic disease management in the community: what needs to be done? BMC Heal Serv Res 2011 11 131 10.1186/1472-6963-11-131 PMC3116473 21619596

[B38] Mair FS May C O’Donnell C Finch T Sullivan F Murray E Factors that promote or inhibit the implementation of e-health systems: an explanatory systematic review Bull World Health Organ 2012 90 357 364 10.2471/BLT.11.099424 22589569 PMC3341692

