# Supplementary material for: Boundaries and e-health implementation in health and social care
Source: BMC Med Inform Decis Mak. 2012 Sep 7;12:100. doi: 10.1186/1472-6947-12-100 (PMC3465217; doi:10.1186/1472-6947-12-100)
Supplement: Additional file 1 — Interview Schedule. [file 1472-6947-12-100-S1.doc]

**Interview Schedule**

**Identifying and understanding the role of boundaries in facilitating or preventing e-health implementation in health and social care: a case study approach.**

**Contextual elements affecting information flow across boundaries**

| **Aims** | 1. In what ways is the SSA intended to help information to flow across structural, professional and geographical boundaries?  2. To what extent do those using the system understand and accept this aim?  3. To what extent has the system helped to improve the communication of information across SPG boundaries?  4. Ideas on how this could best be handled in future projects? |
| --- | --- |
| **Technology** | 1. To what extent does the technology help or hinder information flow across structural, professional and geographical boundaries?  2. To what extent do staff find the technology (a) useful and (b) easy to use? (There is an instrument (based on TAM) to assess response on this dimension).  3. Ideas on how this could best be handled in future projects? |
| **Working processes** | 1. To what extent does the system fit established working processes?  2. To what extent have these changed to fit the system?  3. To what extent has this factor helped or hindered the system meet its aim (information flow across boundaries)?  4. Ideas on how this could best be handled in future projects? |
| **Training** | 1. To what extent were staff given training in how to use the planned system?  2. Did this cover aspects of working with staff in other units, professions or localities?  3. To what extent has training provision helped or hindered the acceptance and use of the system?  4. Ideas on how this could best be handled in future projects? |
| **Finance** | 1. To what extent does using the system fit established financial arrangements for your unit?  2. To what extent have the financial arrangements changed to support use of the system?  3. To what extent has this factor helped or hindered the system meet its aim (information flow across boundaries)? (e.g. does it help you financially?)  4. Ideas on how this could best be handled in future projects? |
| **People**  **(staff, patients, carers?)** | 1. To what extent does the system affect people (e.g. effects of better information across SPG boundaries)?  2. To what extent have people factors changed to fit the system?  3. To what extent has this factor helped or hindered the system meet its aim (information flow across boundaries)?  4. Ideas on how this could best be handled in future projects? |
| **Structural** | 1. To what extent does the system fit the established division of roles and responsibilities in your unit?  2. To what extent have roles and responsibilities changed to fit the system?  3. To what extent has this factor helped or hindered the system meet its aim (information flow across boundaries)?  4. Ideas on how this could best be handled in future projects? |
| **Cultural** | 1. To what extent does the system support or threaten valued features of the unit’s culture?  2. To what extent has your unit’s culture changed to fit the system?  3. To what extent has this factor helped or hindered the system meet its aim (information flow across boundaries)?  4. Ideas on how this could best be handled in future projects? |
| **Other factors** | 1. To what extent have other factors helped or hindered the system meet its aim (information flow across boundaries)? |

**5 Boundaries and care delivery – process**

**Table 2 – Process factors affecting project outcomes**

| **Stakeholders** | 1. To what extent did those responsible for implementation identify the key players with a stake in the project  2. Did they identify their interests, and their ability to influence the outcomes?  3. Ideas on how this could best be handled in future projects? |
| --- | --- |
| **Project vision** | 1. To what extent did they spend time clarifying and communicating a vision for the project?  2. Did they clarify what they expected it to achieve, and how it might get there?  3. Ideas on how this could best be handled in future projects? |
| **Strategic leadership** | 1. To what extent did senior management support the project – such as in public expressions of support, committing adequate resources?  2. Did they establish a clear governance structure? Was there a steering group or project board to oversee the project, and link it to the organisation?  3. How was its membership decided?  4. Ideas on how this could best be handled in future projects? |
| **Terms of reference** | 1. Was there an agreed and published document setting out the terms of reference of the project?  2. Did it cover topics such as why it was being done? who would be involved? how would it be done? by when?  4. Ideas on how this could best be handled in future projects? |
| **Policy** | 1. To what extend has the service matched (met) local or national policy initiatives? |
| **Project team** | 1. Was there a designated project manager, and who did they report to?  2. Who were the members of the team? from which units/professions/localities? Full-time or part-time?  3. Did the team consult with people and groups with an interest in the project?  4. Ideas on how this could best be handled in future projects? |
| **Other factors** | Any other comments on the way in which the project was planned and implemented – what factors helped and hindered it? |
