# Supplementary material for: Boundaries and e-health implementation in health and social care
Source: BMC Med Inform Decis Mak. 2012 Sep 7;12:100. doi: 10.1186/1472-6947-12-100 (PMC3465217; doi:10.1186/1472-6947-12-100)
Supplement: Additional file 2 — Coding scheme for CSO boundaries project. [file 1472-6947-12-100-S2.doc]

**Coding scheme for CSO boundaries project**

**INNER (AND HISTORICAL) CONTEXT**

**1. Aims / purposes of innovation**

1.1 Stated aims of innovation in this unit

1.2 Do people know about, understand and accept these aims?

**2. The characteristics of the innovation**

2.1 Ownership

2.2 Usefulness of technology

2.3 Ease of use (simplicity) of technology

2.4 Congruence and fit between systems

2.5 IT support

2.6 Future models for SSA

2.7 Historical context (past history of IT)

2.8 Suggestions for future

**3. Working processes**

3.1 Fit established working processes, causing extra work

3.2 Hindering work

3.3 Relevance to work

3.4 Access to information

3.5 Security and confidentiality

3.6 Suggestions for future

**4. Financial issues**

4.1 Financial implications

**5. Staff motivation**

5.1 Willingness of staff to use IT in general

5.2 Willingness to use this system to share information

5.3 Changes in skill requirements or development/evolution of skills

5.4 Changes in information they receive about results of work

5.5 Suggestions for future

**6. Structure**

6.1 Difference in local models – workarounds

6.2 Impact on roles and responsibilities

6.3 Suggestions for future

**7. Culture**

7.1 Sharing information with other units or professions – how they relate to each other

7.2 Trust – e.g. of assessments made by others

7.3 Autonomy (e.g. professional or of this unit)

7.4 Staff attitudes to the work which matters most – is this the right thing to be doing – does it fit the culture?

7.5 Readiness/willingness to accept change

**OUTER (AND HISTORICAL) CONTEXT**

8.1 Competing government priorities and aims

8.2 Policy support for this project – inc leadership issues

8.3 Issues of security and information exchange

8.4 Financial policies and practices

8.5 Structural issues – national vs local autonomy; Health Boards/Local Authority arrangements

8.6 IT Infrastructure

8.7 Suggestions for future

**IMPLEMENTATION PROCESSES**

9.1 Training fit for purpose?

9.2 Support from senior managers – e.g. resources

9.3 Project oversight, governance and management

9.4 Champions, leadership – identifiable and skilful?

9.5 Feedback to staff during implementation

9.6 Identification of key stakeholders

9.7 Clarity and communication of project vision and terms of reference (was there a document stating these?)

**BOUNDARIES**

10.1 Structural boundaries affecting the contexts of SSA/CNIS

10.2 Professional/patient boundaries affecting the contexts of SSA/CNIS

10.3 Geographical/location boundaries affecting the contexts of SSA/CNIS

**OUTCOMES**

11.1 Achieved intended aims? Implemented in paper form? Implemented electronically? Staff sharing data?

11.2 Any unintended consequences?

11.3 Variations in use of innovation?

11.4 Economic benefits?

11.5 Patient and/or staff satisfaction?

11.6 Normalisation?

11.7 Suggestions for future

**STAKEHOLDERS**

- 1. Identifying who they are, and their interest in the system
  2. Do they support the system? Have they benefitted from it?

12.3 Suggestions for future
